# Adipose tissue aging is regulated by an altered immune system

**DOI:** 10.3389/fimmu.2023.1125395

**Published:** 2023-02-17

**Authors:** Yi-Xiang Zhang, Min-Yi Ou, Zi-Han Yang, Yu Sun, Qing-Feng Li, Shuang-Bai Zhou

**Affiliations:** ^1^ Department of Plastic and Reconstructive Surgery, Shanghai Ninth People’s Hospital, Shanghai Jiao Tong University School of Medicine, Shanghai, China; ^2^ Key Laboratory of Tissue Microenvironment and Tumor, Shanghai Institute of Nutrition and Health, University of Chinese Academy of Sciences, Chinese Academy of Sciences, Shanghai, China

**Keywords:** aging, adipose tissue, inflammaging, metabolic disease, immune aging

## Abstract

Adipose tissue is a widely distributed organ that plays a critical role in age-related physiological dysfunctions as an important source of chronic sterile low-grade inflammation. Adipose tissue undergoes diverse changes during aging, including fat depot redistribution, brown and beige fat decrease, functional decline of adipose progenitor and stem cells, senescent cell accumulation, and immune cell dysregulation. Specifically, inflammaging is common in aged adipose tissue. Adipose tissue inflammaging reduces adipose plasticity and pathologically contributes to adipocyte hypertrophy, fibrosis, and ultimately, adipose tissue dysfunction. Adipose tissue inflammaging also contributes to age-related diseases, such as diabetes, cardiovascular disease and cancer. There is an increased infiltration of immune cells into adipose tissue, and these infiltrating immune cells secrete proinflammatory cytokines and chemokines. Several important molecular and signaling pathways mediate the process, including JAK/STAT, NFκB and JNK, etc. The roles of immune cells in aging adipose tissue are complex, and the underlying mechanisms remain largely unclear. In this review, we summarize the consequences and causes of inflammaging in adipose tissue. We further outline the cellular/molecular mechanisms of adipose tissue inflammaging and propose potential therapeutic targets to alleviate age-related problems.

## 1 Background

Adipose tissue is a widespread organ roughly divided into white adipose tissue (WAT) and brown adipose tissue (BAT) (1). In addition, beige adipocytes are known to differentiate from progenitors resident in WAT while exhibiting BAT-like morphology and function (1). According to the depots, WAT can also be categorized into subcutaneous adipose tissue (SAT) and visceral adipose tissue (VAT). SAT is located beneath the skin, while VAT surrounds internal organs and is usually found in the mesentery and omentum (2). The function of adipose tissue, including both SAT or VAT, is mainly to store energy, regulate temperature, modulate immune responses, facilitate wound healing and promote tissue regeneration (2).

Aging is considered to be associated with an increasing prevalence of obesity, type 2 diabetes, and other comorbidities (3). These age-related diseases are usually related to adipose tissue, which not only mediates an organism’s adaptation and response to aging but also plays a pivotal role in age-related metabolic dysfunction and longevity (4).

Aging adipose tissue has several characteristics. First, the ratio of VAT to SAT is increased in aged individuals (5). Second, brown and beige fat are reduced during the aging process (3). Third, the function of adipose stem cells and progenitor cells is decreased (6). Fourth, there is an accumulation of senescent cells in aging adipose tissue (7). Finally, but most importantly, aging adipose tissue is linked to a chronic, low-grade inflammation termed inflammaging, which is a central characteristic as it may promote other aging characteristics and influence the overall health status. The thermogenic capability of brown adipose tissue is compromised by proinflammatory cytokines, which may suppress the uncoupled activity of protein-1 (UCP-1) (8). Proinflammatory cytokines also compromise the adipogenic capacity of adipose stem cells (9). Senescent cells promote an inflammatory environment, while proinflammatory cytokines also promote senescent cells (10). Adipose tissue inflammaging is related to an increased body mass, elevated adipocyte size, emerging fragile states and chronic degenerative disorders (11). Therefore, inflammation in adipose tissue may be a potential therapeutic target in antiaging therapy.

The progenitor cell decline phenomenon is observed mainly in aging WAT (3). Similarly, adipokine changes are only observed in aging WAT rather than BAT (3). Briefly, WAT has a more dramatic response to aging than BAT. Thus, we emphasize WAT inflammaging.

In this review, we first discuss the impact of inflammaging on aging adipose tissue and the overall health status and then reveal the factors contributing to adipose tissue inflammation. Furthermore, we frame the alteration in immune cells in aging adipose tissue and the underlying molecular mechanism of inflammaging. Finally, we summarize a potential strategy for antiaging therapy through adipose tissue.

## 2 The impact of inflammaging on adipose tissue

### 2.1 Adipose plasticity

During the process of inflammaging, the plasticity and function of ADSCs are regulated by specific factors. Taha et al. cultured ADSCs treated with TNFα to trigger a strong inflammatory response, and then, deep next-generation mRNA sequencing was performed to evaluate the inflammatory responses of the ADSCs (12). The results showed that the ADSCs exhibited a strong response when exposed to an inflammatory environment. Adipogenesis is also reduced by inflammaging. Liu et al. showed that after the deletion of proinflammatory macrophages in SAT, the differentiation of preadipocytes was upregulated, and the expression of differentiation genes was increased (9). Inflammaging is strongly related to hypoxia (13). Chol et al. found that ADSCs cultured under low-oxygen conditions exhibited a higher proliferative ability, significantly higher basal migration, and reduced lipid production. ADSCs maintain an undifferentiated status in a hypoxic environment, and their potential to differentiate into adipocytes is decreased (14).

### 2.2 Adipocyte remodeling

Adipose tissue inflammaging, as a consequence of proinflammatory immune cell infiltration, may disrupt the recruitment of new adipocytes, leading to adipocyte hypertrophy (15). Adipocyte hypertrophy is closely related to metabolic diseases. Initially, adipocyte hypertrophy is an adaptive reaction to excessive nutrition, which is beneficial in lean objects as it can protect tissues other than adipose tissue from lipotoxicity. However, in some obese or aging patients, the adipocyte buffering ability may be exceeded, reaching the hypertrophic threshold, leading to ectopic lipid deposition in other tissues (16). Adipocyte hypertrophy further exacerbates adipose tissue hypoxia and inflammation, leading to adipose tissue fibrosis and adipocyte apoptosis.

### 2.3 Adipose tissue fibrosis

In aging adipose tissue, the insufficient angiogenic potential, inappropriate ECM remodeling and unresolved inflammation result in adipose tissue fibrosis. An insufficient angiogenic potential leads to hypoxia, which stimulates the transcription of HIF1α (5, 17). On the one hand, the activation of HIF1α inhibits preadipocyte differentiation and initiates adipose tissue fibrosis. On the other hand, HIF1α may induce a change in the cellular redox status, which, in turn, affects enzymes involved in collagen crosslinking and stabilization (18). Fibrosis is characterized by an imbalance in ECM homeostasis, including the balance between ECM production and ECM degradation (19). Studies in aged mice (~30 months of age) demonstrated an increase in WAT collagen staining, indicating more fibrosis in this tissue (20). Two cells play important roles in ECM production, M1-type macrophages and mast cells. Macrophage-inducible C-type lectin (Mincle) production is induced in macrophages through the saturated fatty acid/TLR4/NF-κB pathway and contributes to ECM production. Mast cells can promote fibroblast growth and collagen production by releasing cytokines, chemokines, proteases, etc., eventually leading to ECM production (21, 22). Fibroblasts and macrophages are the primary cell types that mediate collagen internalization and degradation. Studies have shown that an increase in proinflammatory cytokines is linked to the downregulation of metalloproteinase (MMP) expression. MMPs have the ability to cleave ECM components; thus, the increase in proinflammatory cytokines inhibits ECM degradation (21, 22). The size of both VAT and SAT is reduced in aged animals, suggesting that senescence affects lipid processing in adipose tissue, promoting ectopic lipid accumulation.

### 2.4 Ectopic lipid accumulation

Inflammaging caused by proinflammatory immune cells largely damages the function of adipose tissue and eventually leads to adipose tissue fibrosis. As the function and composition of VAT and SAT are affected, ectopic lipid storage is promoted (23). When the dietary buffer cannot be addressed by senescent adipose tissue, lipotoxicity mediated by the ectopic deposition of lipids occurs in the liver and skeletal muscle. Lipotoxicity in these tissues increases the ROS levels and activates serine threonine kinases, such as c-jun N-terminal kinase (JNK), IκB kinase (IKK), and protein kinase C (PKC). These events not only disrupt insulin receptor signaling cascades and promote insulin resistance but also are associated with the development of hepatic steatosis and muscle dysfunction and may trigger the development of sarcopenia (24).

## 3 The impact of adipose tissue inflammaging on the overall health status

### 3.1 Metabolic diseases

Aging-induced proinflammatory cytokines can directly interfere with the insulin signaling pathway in adipocytes (25). In addition, NLRP3 activated by DAMPs mediates chronic inflammation and insulin resistance. The activation of NLRP3 contributes to a higher expression of IL-1β, and IL-1β is a key cytokine in the etiology of type 2 diabetes. Briefly, IL-1β can affect insulin signaling, reduce glucose transporter type 4 (GLUT4) expression, and have proapoptotic effects on β-cells (mediated by the MAPK and NF-κB signaling pathways) (26).

### 3.2 Cardiovascular disease

Adipose tissue can act as an important source of inflammatory mediators, thus promoting systemic inflammaging (27). Chronic inflammation significantly increases the risk of CVD. The proinflammatory cytokines released by inflamed adipose tissue may force perivascular adipose tissue to modify its composition and accelerate atherosclerosis (28). Adipose tissue-derived proinflammatory cytokines, such as IL-1β and TNF, induce the expression of endothelial cell adhesion molecules, which further promote vascular inflammation (29). Exosomes derived from inflamed VAT have been shown to promote the M1 proinflammatory polarization of macrophages and promote atherosclerosis (30).

### 3.3 Cancer

Cancer can arise at a site of inflammation, and a proinflammatory microenvironment is an essential component of cancer. Chronic inflammation can initiate cancer, promote its progression and support its metastatic diffusion (31). Adipose tissue inflammation may also be the driver of cancer (32).

## 4 Factors contributing to adipose tissue inflammaging

### 4.1 Senescent cell accumulation and cell death

A central reason for adipose tissue dysfunction and inflammation during aging is the accumulation of senescent cells. Cellular senescence is a basic aging mechanism that results in organ dysfunction and chronic inflammation (33). Upregulated ROS are the main drivers of adipose tissue senescence. Following ROS upregulation, the DNA damage response (DDR) triggers the p53/p21 signaling pathway, followed by the consequent promotion of the senescence phenotype along with exacerbated TNF-α/IL-6 secretion and β-galactosidase activities. Such DNA injury can eventually pave the path for ATM/p53/p21 upregulation (34). Senescent cells can release several proinflammatory cytokines, which are currently considered hallmark components of the SASP (35). These proinflammatory factors continue to accumulate in tissues as the clearance of senescent cells is compromised during aging (4).

Key essential cells within adipose tissue develop the senescence phenotype. Adipose-derived stem cells (ADSCs) gradually lose the ability to replicate before entering cellular senescence, a state characterized by the upregulation of senescence markers, such as p16^INK4a^, p21^Waf1^ and caveolin-1 (36). p16^INK4a^ is upregulated through p38 MAPK influence, possibly contributing to cellular senescence. In addition, p53 MAPK is involved in age-related ADSC functional transformation. Its activation impairs mitochondrial function. Mitochondrial activities are essential players in maintaining stem cell pluripotency (37). Additionally, ADSCs expand in dimensions, morphology and structural complexity along with the decreased expression of CD105 during the natural aging process (38). Cellular senescence of preadipocytes can progress to widespread shifts in preadipocyte function, such as reduced proliferation, adipogenesis, and exacerbated production of proinflammatory cytokines and extracellular matrix–modifying proteases (39). Senescent preadipocytes also negatively influence adipogenicity within surrounding progenitors and induce them to senesce (34). Cellular senescence is also linked to downregulated PPARγ and the triggering of downstream targets in endothelial cells, suggesting that capabilities, such as responding to fatty acids and promoting lipid transport, are significantly diminished. Moreover, p53 protein upregulation typically occurs in endothelial cells. Senescent endothelial cells lose the capability to discharge endogenous lipid PPARγ ligands, consequently leading to detrimental influences on the differentiation and function of human adipocytes (34). Adipocyte senescence is also induced by elevated DNA damage (40). In addition to adipocyte senescence, adipocyte death occurs upon obesity or during aging (41, 42). Dying adipocytes can attract macrophages, and these macrophages produce various types of cytokines according to the type of cell death. Apoptotic cells establish the production of anti-inflammatory cytokines, whereas necrotic cells establish proinflammatory cytokine production characterized by the secretion of IL-1 by the macrophage population (43). The NOD-like receptor (NLR) family of pattern recognition receptors (PRRs) can sense obesity- or aging-induced signals, such as damage-associated molecular patterns (DAMPs), originating from stressed adipocytes. In macrophages, the activation of NLR activates the NLRP3 inflammasome (44). The inflammasome consists of a multiprotein intracellular complex that develops as a stress-triggering response, leading to the secretion of the proinflammatory cytokines IL-1β and IL-18 (45). IL-1β upregulates IL-2 and TNFα, generating tissue inflammatory activities by triggering cyclooxygenase-2, consequently generating prostaglandin E2, inducible intercellular adhesion molecules and NO (45).

### 4.2 Hypoxia, mechanical stress and obesity caused by adipocyte hypertrophy

Aging leads to adipocyte hypertrophy, even within a single white adipose tissue (WAT) depot, and the cell diameters of different adipocytes can dramatically vary, ranging from less than 20 µm to 300 μm (46). An increased adipocyte size results in decreased oxygen diffusion. In addition, the blood supply to adipocytes is reduced during aging (5). These two factors generate a hypoxic environment in aged adipose tissue, which has been shown to occur with aging as revealed by immunohistochemistry and direct measurements of the interstitial partial pressure of oxygen (47). Hypoxia may induce a reaction mediated by hypoxia-inducible factors (HIFs) to promote angiogenesis and complement oxygen levels (17). However, as the angiogenic potential of ADSCs declines and the expression of VEGF and the density of blood vessels decrease with aging, compensation from the vasculature becomes inadequate. As a result, the reduced oxygen diffusion is aggravated due to insufficient compensation by the vasculature (48).

The expression of HIFs is usually induced by hypoxia, and HIF isoforms have defined, nonredundant functions concerning adipocyte function. Even though HIF-2α expression within adipocytes is beneficial since it exerts key senescence prophylaxis-related metabolic effects, including enhanced vascularization possibilities (49), HIF-1α could perform opposing functions. HIF-1α triggering did not activate typical VEGFα-vascularization responses; rather, HIF-1α induced a collagen-driven profibrotic response that paved the path for maladaptive adipose tissue remodeling and insulin resistance (49). HIF-1α may also reduce the expression of genes in mitochondrial complex IV such that the reduced mitochondrial activity contributes to adipocyte hypertrophy. A previous study showed that the knockout of HIF-1α improved mitochondrial function and reduced adipocyte hypertrophy in middle-aged mice (5). Studies have shown that cultivating adipose tissue in a hypoxic environment induces alterations in gene expression, including the upregulation of inflammation-related genes (50). Furthermore, evidence suggests that the NF-κB signaling pathway is enhanced in hypoxic adipose tissue (13).

Mechanical stress also induces inflammation in adipose tissue. As adipocyte hypertrophy is facilitated by aging, the pathological expansion of the extracellular matrix (ECM) has also been observed, leading to altered mechanical stress (51). Furthermore, mechanical stress is exerted by enlarged lipid droplets within the cell (52). Although the pathways controlled by mechanical stress in adipocytes have not been elucidated, the NFκB signaling pathway may be influenced by mechanical stress *via* the RhoA-Rock signaling pathway (53).

As a metabolic syndrome, obesity frequently develops during old age and is a critical factor associated with adipose tissue inflammaging. Typically, obesity is mediated by adipocyte hypertrophy or hyperplasia. Adipocyte hyperplasia is more metabolically friendly than adipocyte hypertrophy. Adipocyte hypertrophy caused by obesity can result in a hypoxic environment, leading to adipose tissue inflammation. Multiple studies have shown that PDGFα^+^CD9^low^ proadipogenic adipose progenitor cells (APCs) switch to PDGFα^+^CD9^high^ profibrotic progenitor cells when influenced by inflammation, ultimately promoting adipose tissue fibrosis and reducing adipocyte hyperplasia (54). The increased adipocyte hypertrophy with reduced adipocyte hyperplasia contributes to adipose tissue inflammation through aging (55). However, obesity may further worsen immunosenescence by enabling the activation and differentiation of immune cells passing through the adipose tissue microvasculature. Previous research has shown that adipose tissue dysfunction in obesity enables immunological aging along with excessive inflammatory responses (56).

### 4.3 Exogenous and endogenous fatty acids and exogenous lipopolysaccharide

Exogenous and endogenous fatty acids and exogenous lipopolysaccharide (LPS) are capable of inducing inflammation in adipose tissue through the activation of toll-like receptors (TLRs) expressed in both adipocytes and macrophages (57). Gut-derived LPS binds TLR4, while free fatty acids (FFAs) can activate inflammatory signaling through either TLR4 or TLR2 (58).

### 4.4 Dysregulation of immune cells in aging adipose tissue

**Figure 1:**
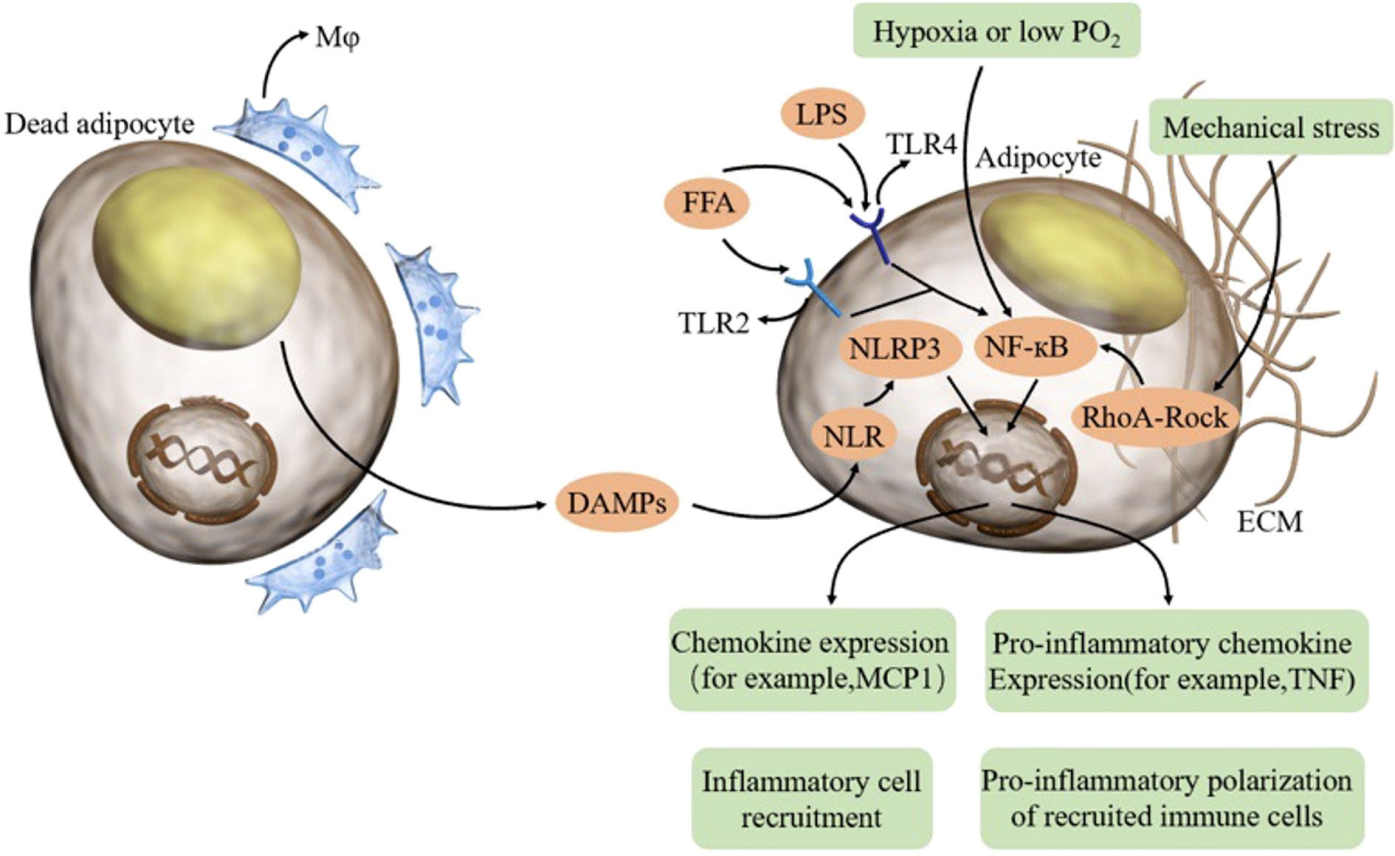
In aging adipose tissue, dead adipocytes induce macrophages and DAMPs, thereby activating the NLRP3 inflammasome in adipocytes. FFA and LPS combine TLR on adipocytes and activate NFκB. Hypoxia and mechanical stress can also activate NFκB. Adipocytes can produce proinflammatory cytokines and chemokines, thus recruiting inflammatory cells and promoting the proinflammatory polarization of recruited immune cells. FFA free fatty acid, LPS lipopolysaccharide, TLR toll-like receptor, DAMP damage-associated molecular patterns, NLR NOD-like receptor.

Immunosenescence, which results in a defective immune response to pathogens and is often coupled with excessive inflammatory activities, is also found in aging adipose tissue. In particular, VAT shows immune cell activation and inflammation compared to other tissues (59). The abnormal activation of immune cells was first detected in WAT depots in middle age and is considered a hallmark of aging (32). In old age, the adipose immune system shifts toward being more unregulated. At this age, various resident regulatory cell populations are dwindling and substituted by inflammatory cells due to phenotypic switching in resident adipose immune cells or the infiltration of inflammatory immune cells from the periphery (60). Prior studies have mainly focused on macrophages, ILCs and eosinophils in adipose tissue. Among these cell types, M2-like macrophages tend to be lost in aged adipose tissue (61). Moreover, there are differential age-related changes between SAT and VAT. For example, the number of eosinophils is substantially reduced in elderly VAT but remains unaffected in SAT (62). Other changes in immune cells are also discussed in this review (**Figure 1**).

## 5 The cellular mechanisms mediating adipose tissue inflammaging

### 5.1 Proinflammatory adipose tissue resident immune cells

#### 5.1.1 Innate immune cells

M1-type macrophages release proinflammatory cytokines, such as TNF-α and IL-1β, to promote adipose tissue inflammation (63, 64). Monocyte chemotactic protein-1 (MCP-1) recruits circulating monocytes to adipose tissue, where they become adipose tissue macrophages (ATMs). LTB4 also promotes macrophage activation and chemotaxis into adipose tissue (65).

An increase in M1-type macrophages and a decrease in M2-type macrophages are observed in aged adipose tissue (66). Inositol-requiring enzyme 1α (IRE1α) induces M1-type macrophage polarization while reducing polarization in M2-type macrophages (67). Similarly, the IRE1α signaling pathway impairs white adipose tissue browning, leading to the development of obesity during aging (68). Further studies have shown that glucose may activate M1 macrophages *via* the ROCK/JNK and ROCK/ERK pathways (69). The Notch 1 signaling pathway is important for M1 polarization and is negatively regulated by the microRNA miR-30 (70). In addition, endoplasmic reticulum (ER) stress signaling helps promote M1 polarization. CHOP, one of its downstream components, is induced by a high-fat diet (71).

Many important signaling pathways are involved in macrophage-induced adipose tissue inflammation. Long-chain saturated fatty acids induce macrophages to produce an inflammatory response through the activation of the JNK signaling pathway (61). TLR signaling is important for macrophage-induced inflammation, acting in conjunction with the Wnt signaling pathway to amplify the release of proinflammatory cytokines (72). Moreover, TLR4 induces the NLRP3 inflammasome in macrophages (73).

Senescent macrophages display increased JNK phosphorylation. The JNK signaling pathway contributes to inflammation and plays a key role in reshaping the metabolic status. Moreover, senescent macrophages show a reduction in SIRT1 expression (74). P38MAPK signaling is increased in senescent macrophages, which is promoted by Arginase-II (Arg-II). In turn, Arg-II reduces Arg-I expression and activity, induces interleukin (IL)-6 expression and secretion, and increases active P38MAPK in aging senescent adipose tissue macrophages (75).

Approximately 80-90% of dendritic cells in adipose tissue are CD11c^+^ conventional DCs (cDCs), and the other dendritic cells are CD123^+^ plasmacytoid DCs (pDCs) (76). cDCs are further subdivided into cDC1s and cDC2s (77). cDC1s are characterized by an activated Wnt/β-catenin pathway, whereas cDC2s show activation of the PPARγ pathway, which suppresses NFκB target genes and leads to the reduced expression of inflammatory genes (61, 78, 79). In a homeostatic state in young and lean subjects, adipocytes can activate the Wnt/β-catenin pathway in cDC1s, leading to the production of the anti-inflammatory cytokine IL-10 (80). In addition, dietary lipids secreted by adipocytes can induce PPARγ signaling in cDC2s, suppressing the activation of inflammatory DCs (81). Both signaling pathways are able to suppress toll-like receptor-4 (TLR4)-induced inflammation in VAT (77). However, when the homeostasis of adipose tissue is compromised, antigen or lipid uptake by ATDCs induces the activation of MAPK signaling, resulting in increased MHC-II expression and cellular maturation (81).

Neutrophils are recruited by the LTB4-BCT1 axis and cytoplasmic phospholipase A2α (cPLA2α). Neutrophils have recently been shown to promote inflammation through the activation of NFκB signaling, and neutrophil activation is closely related to its interaction with adipocytes. Elgazar-Carmon et al. reported that CD11b on neutrophils and ICAM1 on adipocytes mediate their interaction (82). The interaction with adipocytes is critical for the expression of IL-1β *via* NFκB activation in adipose tissue neutrophils (82).

Innate lymphoid cells1/3 are proinflammatory. The activation and proliferation of ILC1 proinflammatory immune cells is promoted by JAK3/STAT5 signaling, while Lnk/Sh2b3 (Lnk) induced by high-fat diet (HFD) signaling suppresses JAK3 (83). IL-15 has also been proven to be important for ILC1 proliferation (83). Similarly, IL-12 could activate adipose ILC1s, leading to the production of IFN-γ and the polarization of M1 macrophages in adipose tissue at the early stages of HFD consumption (84). Studies have shown that ILC1s contribute to not only adipose tissue inflammation but also fibrosis, and this process depends on IFN-γ (85). Furthermore, ILC1s promote fibrogenesis through the activation of CD11c^+^ macrophages and TGF-β1/Smad3 signaling, with TGF-β1/Smad3 activation contributing to persistent and aberrant ECM remodeling in VAT (86). Studies investigating ILC3s are limited, but their proinflammatory and pro-obesity characteristics are well defined. On a mechanistic level, some studies have shown that ILC3s induce obesity through the lymphotoxin/IL-23/IL-22 pathway (87).

**Figure 2:**
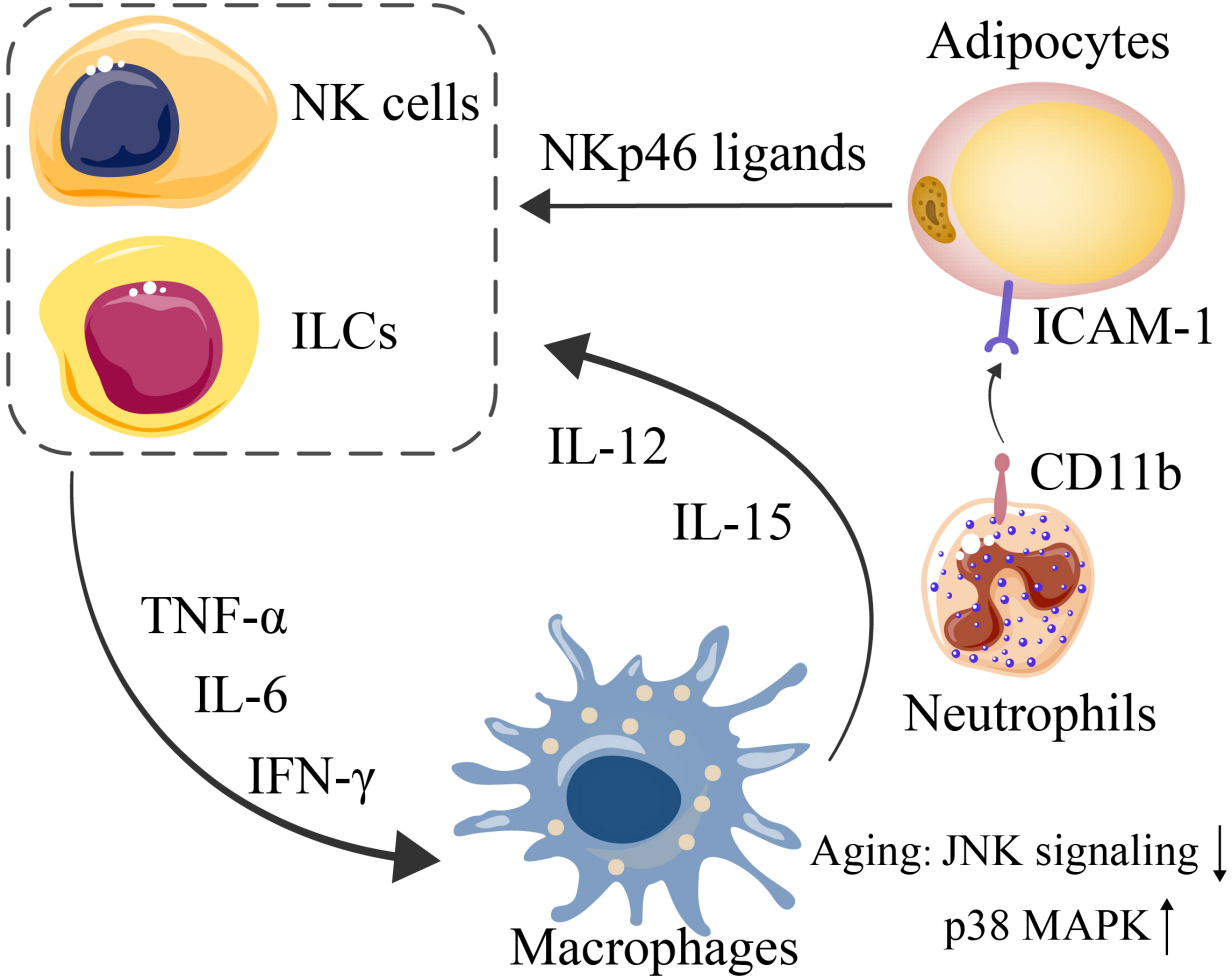
Interactions among adipocytes, NK cells and ILCs (mediated by NKp46 ligands, expressed by stressed adipocytes) lead to further exacerbated expression of IFN-γ, TNF-α, and IL-6. Since macrophages are triggered by such proinflammatory cytokines, IL-12 and IL-15 are consequently secreted by macrophages to induce NK-cell and ILC proliferation. CD11b on neutrophils and ICAM1 on adipocytes mediate their interaction.

Human adipose tissue-resident NK cells are predominantly CD56^bright^CD16^-^ NK cells (88, 89). NK cells and other ILC1s in adipose tissue are often stimulated by proinflammatory factors, such as IL-12, IL-15, and NKp46 ligands produced by macrophages and stressed adipocytes, to promote the production of IFN-γ, TNF-α and IL-6, thereby aggravating the inflammatory response in adipose tissue (**Figure 2**).

#### 5.1.2 Adaptive immune cells

T cells are easily affected by aging and tend to polarize into a proinflammatory phenotype. T cells from both SAT or VAT exhibit senescent features, including the loss of CD28 and expansion of CD44^+^CD62^-^ memory T cells. With aging, there are increases in the numbers of CD4^+^ and CD8^+^ T cells (90–92).

CD4^+^ T cells are activated by MHC-II, and the expression of MHC-II on adipocytes is induced by free fatty acids possibly *via* the activation of JAK and STAT1, which may further activate CIITA, a prime regulator of MHC-II (93). MHC-II is also expressed on other APCs, and in macrophages, CD40 signaling in adipose tissue macrophages regulates MHC II and CD86 expression to control the expansion of CD4^+^ T cells (94). Subtype changes among CD4^+^ T cells represent an important modulator mechanism in different metabolic diseases. Adipose tissue contains a large amount of TGFβ and IL-6. The TGFβ/Smad pathway limits Th1 and Th2 differentiation through the downregulation of T-bet/GATA-3 expression, leading to increased Th17 differentiation (95). IL-6-induced STAT3 can promote the differentiation of Th17 cells (96). In addition, MAP4K4 in T cells can phosphorylate TRAF2, thus downregulating the expression of IL-6, leading to the inhibition of Th17 differentiation and preventing insulin resistance (95). Adiponectin has been shown to directly enhance Th1 differentiation by activating the p38-STAT4-T-bet axis (97). Nevertheless, studies have shown that the PD-L1:PD-1 axis can inhibit Th1 proliferation and promote Th2 polarization, thus limiting the inflammation induced by T cells (98). Regarding CD8^+^ T cells, it has been shown that CD40-TRAF2/3/5/6 signaling is important for CD8^+^ T cells to promote adipose tissue inflammation (99).

Recent studies have shown that the numbers of γδT cells are increased in adipose tissue during aging and contribute to adipose tissue inflammation (100). However, γδT cells also play a role in maintaining adipose tissue homeostasis through adipose tissue browning. Studies have shown that γδT cells and IL-17F upregulate the expression of TGFβ1 in adipocytes by signaling through IL-17RC. In turn, adipocyte-derived TGFβ1 promotes sympathetic innervation, promoting adipose tissue browning (101).

**Figure 3:**
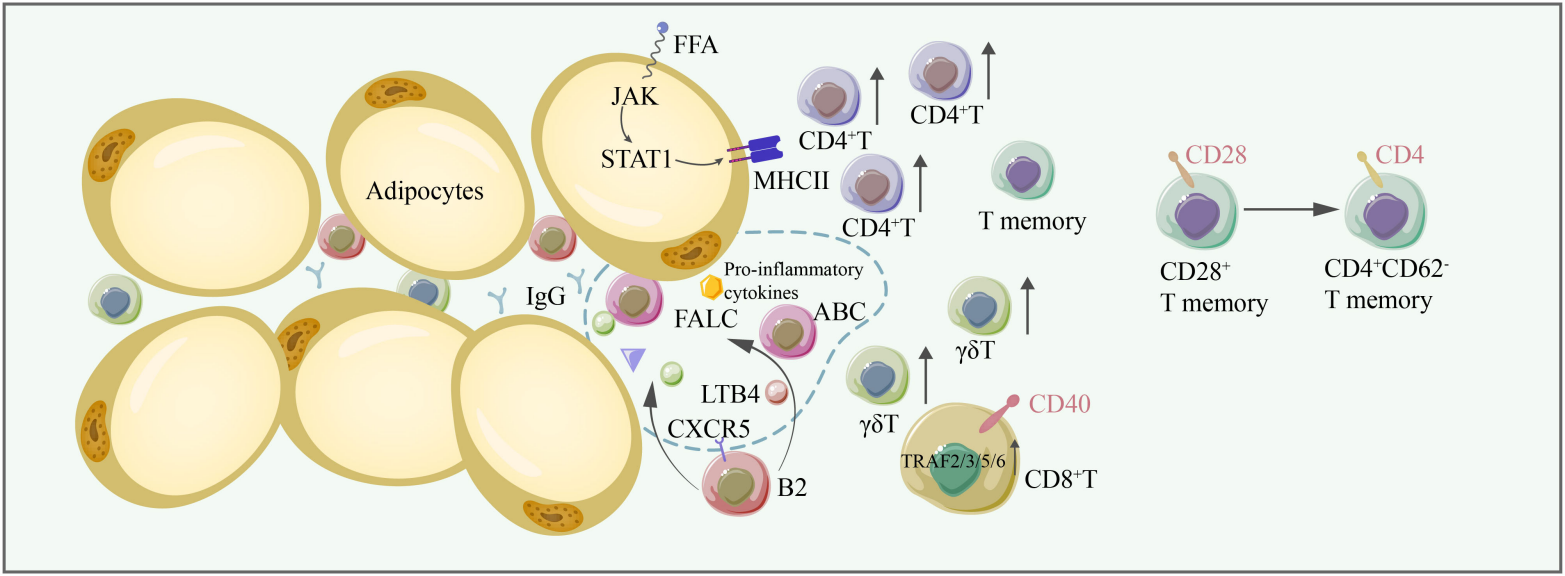
In aging adipose tissues, the number of conventional T cells, γδT cells, increases, thus contributing to adipose tissue inflammation. CD28+ T memory cells are converted to CD4+ CD62- memory T cells. FFA induces the expression of MHC-II through the JAK/STAT1 signaling pathway, facilitating antigen presentation to CD4+ T cells. CD40/TRAF2/3/5/6 signaling is critical for CD8+ T-cell-induced inflammation. B2 cells are recruited to FALC by LTB4, and CXCR5 on B2 cells is important for their recruitment to FALC. Some B2 cells become ABCs. B2 cells and ABC are proinflammatory, producing IgG and exacerbating inflammation in adipose tissue. FFA free fatty acid, FALC fat-associated lymphoid cluster, LTB4 leukotriene B4, CXCR5 C-X-C chemokine receptor type 5.

B2 cells accumulate in adipose tissue prior to T cells. LTB4R1 expression on B2 cells is important for B2 cell chemotaxis. B-cell recruitment to fat-associated lymphoid clusters (FALC) and VAT is also mediated by the CXCR5 signaling axis (102). IgG derived from B2 cells can contribute to adipose tissue inflammation. Aging induces the accumulation of B2 cells in VAT, and B2 cells become more inflammatory once they infiltrate VAT. The following two B-cell types emerge in aging mice: aged adipose B cells (AAB) and aging-related B cells (ABC). The percentage of follicular B cells is reduced, while ABC is increased in VAT in aging mice. Further studies have shown that ABC originates from follicular B cells. AAB primarily reside in FALC, and the expansion of AAB along with their secretion of proinflammatory cytokines and monocyte-recruiting chemokines further aggravate adipose tissue inflammation (103). The activation of NLRP3 is essential for increasing the AAB numbers, FALCS number, and lipolysis by upregulating IL-18 and the IL-1β/IL-1βR axis (104). IL-1R signaling is critical for AAB proliferation (103). Furthermore, activated monocytes can convert innate B1a cells into 4BL cells (4–1BBL^+^ B1a cells), inducing cytolytic CD8+ T cells and insulin resistance in elderly individuals (105) (**Figure 3**).

### 5.2 Anti-inflammatory adipose tissue resident immune cells

#### 5.2.1 Innate immune cells

M2-type macrophages and type 2 cytokine signaling are crucial for adipose tissue homeostasis (63). The JAK/STAT pathway is known to control macrophage biology. STAT1 induces M1 macrophage polarization, while STAT6 mediates IL-4a signaling and regulates many M2 signature genes (106). PPAR-γ promotes primary human monocytes to differentiate toward an M2 phenotype (107). Macrophage PPARγ inhibits IFNβ production by interfering with the IRF3-mediated transcription of IFNβ. Interactions between PPARγ and STAT6 facilitate the induction of PPARγ-regulated genes (108). PPARγ activation in murine macrophages induces miR223 expression by binding upstream of miR223, which, in turn, inhibits the expression of NFAT-5 and RAS p21 protein activator 1, promoting the development of an anti-inflammatory M2-like phenotype (109, 110). Several members of the CCAAT-enhancer-binding protein (C/EBP) family play important roles in macrophage activation. While C/EBPσ was shown to induce M1-like proinflammatory responses, cAMP response element-binding protein (CREB) inhibits the expression of M1-associated genes through p38-mediated induction of IL-10 (111). Interferon regulatory factors (IRFs) also play important roles in macrophage polarization. IRF5 was reported to promote M1 macrophage polarization, while IRF3, 4, 6, and 9 promote M2 macrophage polarization (110). The metabolic regulators SIRT and AMPK perform important functions in macrophage polarization. The adipocyte-specific knockout of SIRT accelerates the recruitment of macrophages and polarizes cells into the M1 type. Moreover, the levels of SIRT1 are inversely correlated with BMI (112). AMPK can inhibit proinflammatory responses in macrophages and promote macrophage polarization into an anti-inflammatory phenotype. AMPK can interfere with inflammation by inhibiting NF-κB signaling through the regulation of downstream mediators of NF-κB signaling, including SIRT1, PGC-1α, p53, and Forkhead box O (FoxO) factors (61). AMPK can enhance SIRT1 expression by increasing the NAD/NADH ratio (113).

Although cDC2s are proinflammatory, cDC1s play pivotal roles in circumventing obesity development during aging through a reduction of inflammatory activities, affecting the iNKT and NK-cell abundance. The presence of cDC1s is not typically affected by aging, while there are increasing numbers of cDC2s in VAT in aged mice (114). Regulatory DCs in adipose tissue have been shown to play an important role in the prevention of adipose tissue inflammation as they inhibit specialized autoreactive T cells through perforin (115). However, recent studies have shown that regulatory DCs are reduced with aging, resulting in greatly reduced immune tolerance in adipose tissue (115).

Eosinophils, identified by the CD45^+^CD14^-^CD16^-^CD117^-^Siglec-8^+^CD66b^+^ phenotype, play a key protective role in the innate immune response (116). One of their functions includes the induction of M2-type macrophages through the production of IL-4 (117). Eosinophils are also critical immune cells involved in adipose tissue browning that may be capable of driving the activation of adipocyte beiging through paracrine signaling mechanisms (118). The FGF21-CCL11 axis is critical for type 2 immune cell activation and the beiging of SAT, which is critical for adipose tissue homeostasis (119). FGF21 is usually induced in adipocytes by cold exposure through cyclic AMP-mediated activation of protein kinase A and p38 MAPK, which, in turn, phosphorylates the transcription factor ATF2 for the transactivation of the FGF21 gene promoter (120). Then, FGF21 stimulates CCL11 production through the KLB-ERK1/2 signaling cascade. CCL11 further recruits eosinophils to SAT and contributes to the recruitment of M2-type macrophages (121). Furthermore, iNKT cells may also induce FGF21 (122). IL-4 is expressed and secreted by eosinophils and binds its receptor IL-4R on adipocytes, where it activates the PI3K-AKT and MAPK/ERK signaling pathways, promoting adipocyte beiging. IL-13 produced by ILC2s has the same properties as IL-4 (118). The IL4/13-IL4Rα-STAT6 pathway is required for the biogenesis of functional beige fat (118). Limited studies have shown that the distribution and function of ATEs change throughout aging, leading to defects in adipose tissue homeostasis and low-grade chronic inflammation. First, the migration and regulatory functions of ATEs significantly decrease (123). Second, the level of CCL11 (eotaxin-1), a potent ATE chemoattractant, systemically increases with aging (124). However, CCL11 is negatively correlated with the distribution of ATEs. Third, the decreased ATE/ATM ratio in elderly individuals leads to significant increases in IL-6, IL-1β, and adipocyte hypertrophy, which are closely related to adipose tissue inflammation (125).

ILC2s (CD45^+^Lin^–^CD127^+^CD161^+^CRTH2^+^) play essential roles in adipose tissue homeostasis by maintaining eosinophils and M2-type macrophages (126). However, a significant loss of ILC2s is observed during aging. Moreover, IL-33 normally stimulates the expansion of ILC2s and promotes an anti-inflammatory profile. The binding of IL-33 to the IL-1 receptor–related protein ST2 coupled to an IL-1RAcP (IL-1 receptor accessory protein) unit leads to the release of NFκB (127). Activated NFκB promotes the transcription of GATA binding protein 3 (GATA3), ST2, and consequently, IL-5 and IL-13, leading to the activation of ILC2s and a type 2 immune response (128). IL-33, however, can also stimulate the accumulation of pathogenic ILC2s, which leads to adipose tissue inflammation.

#### 5.2.2 Adaptive immune cells

Aging leads to an increase in Tregs in VAT that continues as mice age (129). IL-33 efficiently induces the process (130). Middle-aged to old mice exhibit a 7- to 11-fold increase in adipose tissue Tregs compared to young mice, with these cells accounting for more than 50% of all CD4^+^ T cells in adipose tissue (131). Interestingly, Treg depletion in young mice may increase the levels of several inflammatory markers in adipose tissue (132). However, the depletion of adipose tissue Tregs in aging mice does not significantly enhance systemic or tissue inflammation (131). Tregs play a protective role in adipose tissue homeostasis. Studies have shown that inducible T-cell costimulator (ICOS) signaling negatively regulates the recruitment of Tregs *via* PI3k-dependent mechanisms. In contrast, the absence of ICOS signaling enhances the recruitment of Tregs and increases the expression of CCR3 (133). The maintenance of Tregs in lean adipose tissue depends on PPARγ, IRF4, BATF, Blimp1, and IL-33 signaling, with PPARγ being a prime activator of Treg accumulation (98). The combination of PPARγ and Foxp3 can further enhance and promote the expression of a Treg-specific transcriptome (134). PPARγ^+^ Tregs can be affected by IFN-γ released by pDCs and eventually become apoptotic (135). The IL-33 receptor ST2 plays important roles in Tregs as follows: insulin signaling can drive the transition of CD73^hi^ST2^lo^ into CD73^lo^ST2^hi^ adipose Treg cell subsets through the HIF-1α–Med23–PPARγ axis (136). Furthermore, PLZF^+^ γδT cells were shown to induce the abundance of IL-33 and ST2 through enhanced IL-17A expression (129). The IL-33/ST2 axis is also important for the differentiation of Tregs, and the downstream signaling of IL-33 is mediated by MyD88 (137). In addition to IL-33, a recent study showed that IL-2 can upregulate hydroxy-prostaglandin dehydrogenase (HPGD) in Tregs through JAK3/STAT5 signaling, and HPGD can further inhibit conventional T cells through PPARγ, thus maintaining the homeostasis of adipose tissue (98). Furthermore, KLF10 is an important protein regulating the differentiation and chemotaxis of Tregs. A decrease in KLF10 in obese subjects impairs the PI3K-Akt-mTOR signaling pathway in Tregs, leading to the impaired migration and decreased accumulation of Tregs in adipose tissue (138). AKT signaling, however, is reported to reverse the ability of Treg cells to inhibit TNF-α production by macrophages (98). TCR signaling is another important signaling pathway for the induction of Treg precursors in VAT (139). Similarly, Stat6/Pten signaling plays an important role in the induction of Tregs into adipose tissue during cold stimulation (134). Tregs, however, can also play a negative role in adipose tissue homeostasis by producing IL-10, which is mediated by Blimp-1. IL-10 can directly suppress thermogenesis in adipocytes through a STAT3-dependent signaling pathway (140).

Invariant natural killer T cells (iNKT) play a protective role in adipose tissue homeostasis. iNKT produce anti-inflammatory cytokines, such as IL-4 and IL-10, and regulate the function of M2 macrophages. iNKT can also upregulate the expression of FGF21 in both BAT and SAT, which drives the activation of BAT and browning of WAT (141). In addition, in obese subjects, iNKT cells can help eliminate hypertrophic adipocytes while promoting adipogenesis through the FAS/FASL pathway, contributing to adipose tissue homeostasis (142). However, recent studies have identified heterogeneity in iNKT subtypes; NK1.1^-^ iNKT cells have upregulated IRE1α/XBP1 signaling, exerting an anti-inflammatory profile, while NK1.1^+^ iNKT cells can release IFNγ, promoting adipose tissue inflammation (143). iNKT cells decrease in aging adipose tissue (90–92).

**Figure 4:**
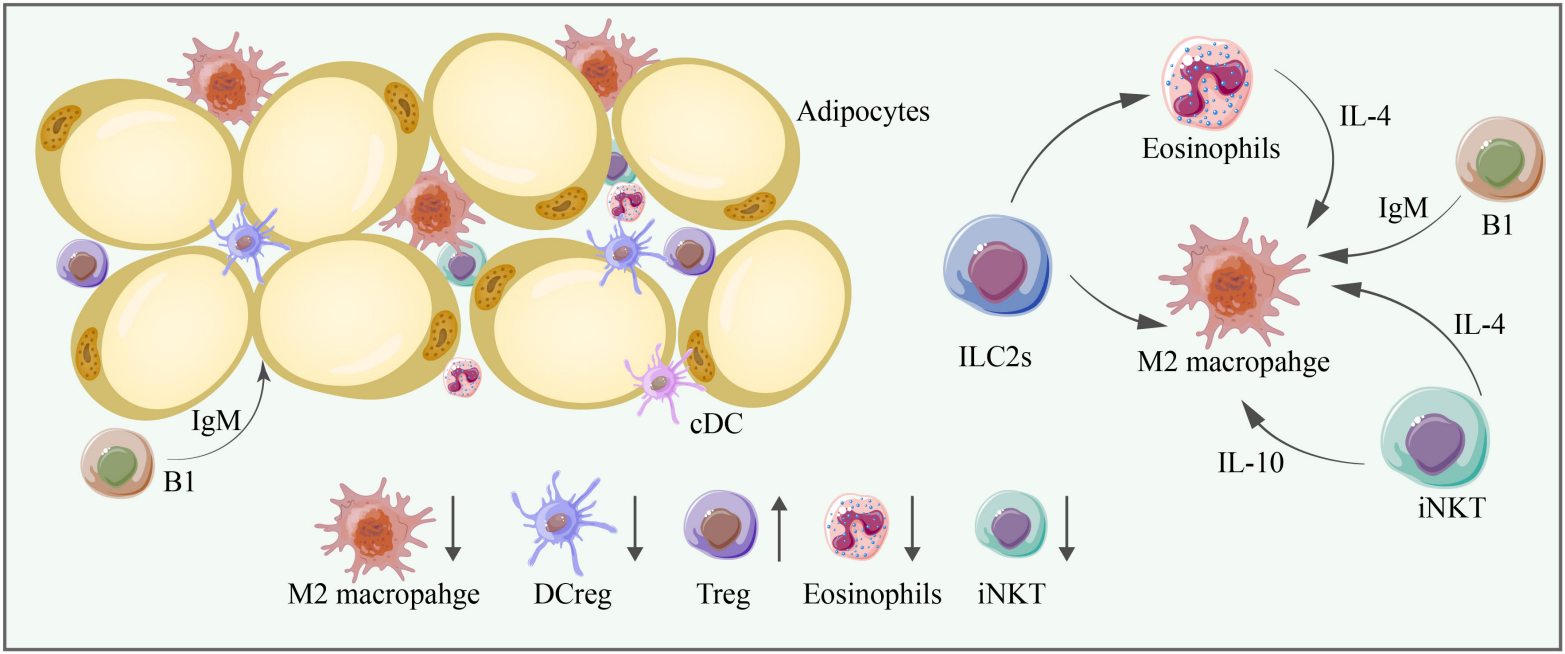
M2 macrophages, DCregs, Tregs, eosinophils, and iNKT cells are significant anti-inflammatory immune cells in adipose tissue, and their abundance is mostly decreased in aging adipose tissue. Cell–cell interactions are essential for adipose tissue homeostasis. ILC2s can promote the proliferation of eosinophils and M2 macrophages, and eosinophils can promote the proliferation of M2 macrophages through IL-4. iNKT cells can promote the proliferation of M2 macrophages through IL-4 and IL-10. B1 cells produce IgM, which exerts anti-inflammatory effects and promotes the proliferation of M2 macrophages.

IgM derived from B1 cells blocks inflammation. IgM antibodies can clear self-antigens and play a regulatory role by promoting B-cell tolerance. They can also induce M2 macrophage polarization in adipose tissue (102) (**Figure 4** and **Tables 1**, **2**).

**Table 1:** Immune cells and their functional implications in aging.

Immune cell types	Marker gene (mouse)	Functional implications	References
Macrophages	Apoe, F13a1, Rbpj, Mrc1, Mmp12, Mctp1, Gpnmb, Apobec1, Atp6v0d2, Cd84	1) An increase in M1-type macrophages and a decrease in M2-type macrophages are observed in aged adipose tissue. 2) Senescent macrophages display increased JNK phosphorylation and P38MAPK signaling and a reduction in SIRT1 expression.	Chung, Nati et al. (63) Varghese, Griffin et al. (144), Hildreth, Ma et al. (64), Albright, Dunn et al. (145),
Dendritic cells	Wdfy4, Cd74, H2-Ab1, H2-Eb1, Plbd1, AC163354.1, H2-Aa, Cbfa2t3, Tbc1d8, Flt3	1) cDC1s are characterized by an activated Wnt/β-catenin pathway, whereas cDC2s show activation of the PPARγ pathway. 2) cDC1s play pivotal roles in circumventing obesity development during aging. 3) cDC2s increased in aging adipose tissue.	Mráz, Cinkajzlová et al. (76), Soedono and Cho (77) Sundara Rajan and Longhi (146)
Neutrophils	S100a9, Csf3r, S100a8, Il1b, Cd300lf, Cxcr2, Trim30b, Retnlg, Sell, Nlrp12	1) Neutrophil recruitment is mediated by cPLA2α and the LTB4-BCT1 axis. 2) Neutrophil activation is closely related to its interaction with adipocytes.	Hadad, Burgazliev et al. (147), Tam, Chan et al. (148),
Eosinophils	Ccr3, Ccl11, Prg2, Epx, Il15, Il4, Mpo, Cebpa, Cebpe, Etv6	1) Eosinophils are necessary for adipose tissue homeostasis. 2) Most immune regulatory characteristics of ATE are achieved through IL-4.	Wu, Molofsky et al. (117), Brigger, Riether et al. (125),
ILCs	Csf2, Arg1, Cd3e, Cd28, Il12rb2, Alox5, Pparg, Il23r, Chad, Tnfrsf21	1) ILC2s contribute to AT hemostasis by maintaining eosinophils and M2 macrophages. 2) ILC1s contribute to the polarization of macrophages toward an M1-like phenotype. 3) ILC3s induce inflammation through the lymphotoxin/IL-23/IL-22 pathway.	Brüggen, Strobl et al. (126), Hildreth, Ma et al. (64), Suffiotti, Carmona et al. (149),
NK cells	AC140209.1, Skap1, Klrk1, Gzma, Ncr1, Kcnq5, Ripor2, Prkcq, Stat4, Txk	1) NK-cell-derived TNF is a primary driver of ATM activation. 2) NK cells are stimulated by proinflammatory factors, such as IL-12, IL-15, and NKp46 ligands produced by macrophages and stressed adipocytes to promote the production of IFN-γ, TNF-α and IL-6	Fernø, Strand et al. (25),
T cells	Skap1, Inpp4b, AC140209.1, Themis, St6galnac3, Prkcq, Tox, Bcl11b, Cd247, Arhgap15	1) Polarize into a pro-inflammatory phenotype. 2) Loss of CD28 and expansion of CD44^+^CD62^-^ memory T cells. 3) Increases in the numbers of CD4^+^ and CD8^+^ T cells and a decrease in the number of iNKT cells. 4) Treg is elevated during aging. 5) γδT cells are increased in adipose tissue during aging	Pan, Yao et al. (150), Bapat, Suh et al. (131), Kohlgruber, Gal-Oz et al. (129),
B cells	Bank1, Agbl1, Ighm, Ripor2, Pax5, Tmem163, Aff3, Ralgps2, Inpp4b, Ikzf3	1) B1 accumulate in aged mice. 2) Activated monocytes convert innate B1a cells into 4BL cells. 3) B2 cells produce pro-inflammatory igG and cytokines	Reyes-Farias, Fos-Domenech et al. (105),. Carter, Miard et al. (151),

**Table 2:** Comparison of subcutaneous and visceral adipose tissue inflammation.

	SAT	VAT	Reference
Macrophages	1. Lipid-rich CD11c^+^ATMs appear late 2. Less ATMs form crown-like clusters surrounding dying adipocytes	1. Lipid-rich CD11c^+^ATMs appear early 2. More ATMs form crown-like clusters surrounding dying adipocytes	Muir, Kiridena et al. (152) Michailidou, Gomez-Salazar et al. (153),
Eosinophils	Eosinophil numbers remain unchanged during aging	Eosinophil numbers decrease during aging	Wu and Ballantyne (154)
Mast cells	1. The ratio of MC_TC_ and MC_T_ is higher in lean individuals 2. Lower TNF levels present in mast cells	1. The ratio of MC_TC_ to MC_T_ is lower in lean individuals 2. Higher TNF levels present in mast cells	Żelechowska, Agier et al. (155), Altintas, Nayer et al. (156),
NK cells	Contains less inflammatory NK cells	Contains more inflammatory NK cells	Bonamichi and Lee (157)
T cells	1. Higher numbers of Foxp3^+^ Treg 2. The number of T cells decreases during aging	1. Lower numbers of Foxp3^+^ Treg 2. The number of T cells increases during aging	Wang and Wu (141) Pan, Yao et al. (150),
B cells	1. B2 cells do not accumulate during aging 2. The ratio of B1 to B2 cells is much lower 3. FALCs cannot be detected	1. B2 cells accumulate during aging 2. The ratio of B1 to B2 cells is much higher 3. FALCs can be detected	Srikakulapu and McNamara (102) Carter, Miard et al. (151),

## 6 The molecular mechanisms mediating adipose tissue inflammaging

### 6.1 JAK/STAT signaling pathway

Over the past two decades, the JAK/STAT signaling pathway has been shown to play vital roles in the regulation of lipid metabolism, glucose metabolism, and adipokine secretion in adipose tissue (158). While early research mainly focused on the role of JAK/STAT signaling in adipogenesis, subsequent studies further demonstrated a role in mediating inflammation in adipose tissue (159). The JAK/STAT signaling pathway mediates the activation of peripheral blood B cells and promotes the activation and proliferation of ILC1s in adipose tissue (83). Adipocytes also express several receptors for JAK/STAT-activating cytokines and hormones, including immune cytokines acting in a paracrine manner to induce JAK/STAT signaling in adipocytes (160). For example, IFN-γ can induce JAK1/STAT1 signaling in human adipocytes and promote inflammation and insulin resistance (161). STAT proteins heterodimerize with phosphorylated IRFs to activate the expression of inflammatory gene signatures (162). Ligands, including IL-6 and platelet-derived growth factor receptors (PDGFRs), also signal through the JAK/STAT signaling pathway (163). Oncostatin M (OSM) is a cytokine expressed in immune cells in adipose tissue, while its receptor OSMR is expressed on adipocytes (164). The loss of OSMR expression is accompanied by decreased STAT5 phosphorylation in adipocytes and a reduction in the expression of proinflammatory cytokines and chemokines (165). Recent studies have reported crosstalk between the JAK/STAT and TGFβ signaling pathways. Transient inflammation caused by lipolysis leads to the upregulation of IL-6 and activation of JAK/STAT3 signaling in adipose progenitors, alleviating the inhibitory effect of TGFβ on adipogenic lineage commitment and thermogenic beige adipocyte differentiation (166).

### 6.2 Wnt/β-catenin and PI3K/AKT signaling pathways

Wnt signaling plays pivotal roles in modulating the adipose tissue microenvironment. Wnt signals can alter important steps of insulin utilization within cells, leading to the progression of insulin resistance (167). The binding of Wnt ligands to their receptors activates the Wnt pathway and causes adipose tissue inflammation, obesity, and glucose homeostasis. Wnt signaling can be further divided into canonical and noncanonical Wnt signaling. Noncanonical Wnt ligands, such as Wnt5a, are expressed in adipocytes at high levels when the diet consists of fatty substances, which contributes to obesity-associated inflammation (168). Wnt5a can also activate JNK and promote insulin resistance (169). The Wnt signaling pathway is activated in cDC1s, leading to the production of the anti-inflammatory cytokine IL-10 (80). However, TLR signaling acts in conjunction with the Wnt signaling pathway to amplify the release of proinflammatory cytokines in macrophage-induced inflammation (72). PI3K signaling is also closely related to chronic inflammation and insulin resistance. Inflammation or other stress stimuli block the PI3K signaling pathway downstream of the insulin receptor through the activation of several serine/threonine kinases, contributing to insulin resistance in adipocytes (170). PI3K signaling and its downstream effectors, including protein kinase B (AKT), help preserve beneficial macrophage subpopulations (75). However, the PI3K/AKT-mediated anti-inflammatory effects are inhibited in aging adipose tissue DCs (171). PI3Kγ can promote neutrophil infiltration.

### 6.3 NF-κB signaling pathway

The NF-κB and JNK signaling pathways are two central mediators of the inflammatory response in adipose inflammation. Specific dietary saturated fatty acids induce the expression of MCP-1 and SAA in adipocytes through the activation of the NF-κB and ROS pathways (172). The activation of NF-κB signaling increases the expression of TNF-α, IL-6 and MCP-1, leading to serine phosphorylation of IRS-1, thus blocking insulin signaling downstream of insulin receptors (26). The transcription of NF-κB is mainly controlled by the phosphorylation of inhibitor of NF-κB (IκB) by the upstream IκB kinase (IKK). Canonical IKKs, including IKKα and IKKβ, phosphorylate IκB and other subunits of NF-κB to induce the expression of NF-κB target genes. IKKα phosphorylates IRS-1 and, thus, induces insulin resistance (153). Panahi et al. found that IKKβ deficiency in adipocytes prevents the expression of proinflammatory cytokines, such as IL-6 and TNF-α, induced by free fatty acids. In contrast, the activation of IKKβ inhibits the expression of anti-inflammatory cytokines, such as adiponectin and leptin (173). In addition to canonical IKKα and IKKβ, two noncanonical IKKs, IKKϵ and tank-binding kinase 1 (TBK1), play roles in adipocyte biology (174). Proinflammatory cytokines, such as TNF-α, activate TBK1, which attenuates adipose tissue inflammation by repressing the atypical NF-κB pathway. In this pathway, NF-κB-inducing kinase (NIF) phosphorylates Ser176 to activate IKKα, which, in turn, activates the RelB (NF-κB2) precursor p100, inducing its maturation. This pathway induces the expression of target genes, such as CCL2, and promotes infiltration by macrophages. TBK1 can further inhibit the metabolic regulator AMPK, thus promoting inflammation (175). Moreover, NFκB activation in adipose tissue neutrophils is important for its expression of IL-1β (82). Activated NFκB also promotes the activation of ILC2s and a type 2 immune response (128). DR3, a recently defined receptor expressed on ILC2s, can also induce and activate ILC2s through NFκB pathways (176).

### 6.4 MAPK signaling pathway

The following two distinct MAPK signaling pathways play important roles in adipocyte inflammation: the p38 pathway and the JNK pathway. JNK signaling is activated in adipocytes in obese humans and mice and promotes insulin resistance through the phosphorylation of IRS, thereby decreasing PI3K/PKB signaling downstream of insulin receptors (153). The activation of JNK can further activate the transcriptional regulator AP-1 and induce the expression of inflammatory genes, such as IL-6 and TNF, inhibiting insulin signaling and causing insulin resistance (25). In contrast to JNK, p38α activity is reduced in obese mice, with concurrent activation of other p38 isoforms, including p38g and p38d (177). The p38 pathway is activated in adipose tissue immune cells, such as macrophages, to promote the production of inflammatory cytokines and the recruitment of monocytes, leading to adipose tissue inflammation (178). Activated p38MAPK signaling in mast cells can also contribute to metabolic dysfunction in SAT (179). The p38 pathway is also implicated in adipocytes as the inhibition of p38 in adipocytes results in a decreased secretion of TNF-α-induced IL-6 (180). JNK plays a role in lipolysis induced by TNF-α, while the role of p38 in this process needs further elucidation (181). When MAPK signaling is activated in DCs, the expression of MHC-II and cellular maturation are increased (81).

### 6.5 AMPK signaling pathway

**Figure 5:**
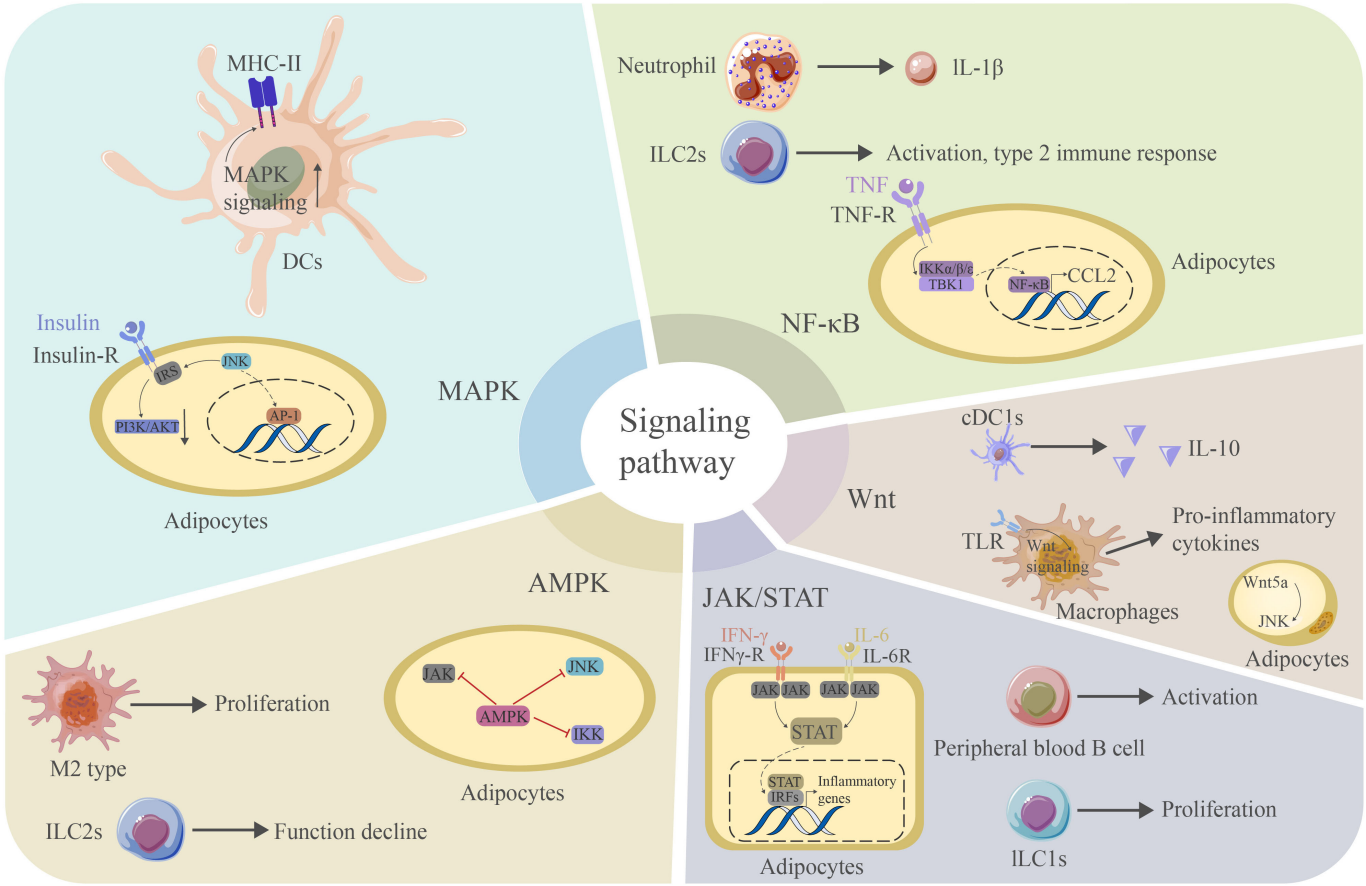
The MAPK signaling pathway is critical for the expression of MHC-II in DCs. JNK signaling can interact with IRS to reduce PI3K/AKT signaling and contribute. JNK can further promote AP-1 production and promote inflammation in adipose tissue. The NF-κB signaling pathway mediates the production of IL-1β in neutrophils and the activation of ILC2s. This pathway is also essential for the adipocyte response to TNF and the production of CCL2. Wnt signaling mediates IL-10 production in CDC1s and binds TLR to produce proinflammatory cytokines in macrophages. Wnt5a can induce JNK in adipocytes. The JAK/STAT signaling pathway is essential for activating peripheral blood B cells and the proliferation of ILC1s. This pathway is also critical for the adipocyte response to IFN-γ. STATs can further bind IRFs to induce the production of proinflammatory cytokines. The AMPK signaling pathway mediates the proliferation of M2-type macrophages and blocks the proinflammatory signaling pathway in adipocytes. However, it may cause a decrease in the function of ILC2s. AP-1 activator protein 1, CCL2 chemokine ligand 2, TLR toll-like receptor, IRF interferon regulation factor.

The final signaling pathway we aim to discuss here is the AMPK pathway, an important anti-inflammatory signaling pathway in adipose tissue. AMPK signaling can inhibit the synthesis of proinflammatory cytokines, including IL-6 and IL-8, in adipocytes, while a deficiency in AMPK leads to an increased production of proinflammatory cytokines. The knockdown of AMPKα1 in 3T3-L1 adipocytes leads to an increase in the mRNA levels of the proinflammatory cytokines TNF-α, IL-1β and MCP-1 in response to FA treatment (182). In a recent study, the following more detailed mechanism was elucidated: AMPK might block a proinflammatory IL-6 trans-signaling mechanism involving IL-6/soluble IL-6R that can stimulate JAK, thus influencing the JAK/STAT3 signaling pathway in adipocytes. Furthermore, AMPK can block IL-1β-stimulated IRAK-4, thus influencing the JNK signaling pathway in adipocytes. Moreover, AMPK can block TNF-α-stimulated IKKβ, thus influencing the NF-κB signaling pathway in adipocytes (183). Thus, AMPK plays a critical role as a master regulator of inflammation by regulating several inflammatory pathways (82). In addition, AMPK can promote M2-type macrophage polarization (61) and compromise the function of ILC2s by interacting with the NFκB pathway (184) (**Figure 5**).

## 7 Adipose tissue as a therapeutic target in aging

In a recent targeted pathway proteomics study, the researchers revealed that aging has tissue-specific effects on WAT in mice, with alterations in metabolic and inflammatory pathways, suggesting that WAT could be critical for an organism’s adaptation and response to aging (185). It has also been shown using bulk RNA-sequencing of 17 organs and plasma proteomics at 10 ages across the mouse lifespan that immune cell activation was first detected in white adipose depots during middle age (186). Many classic mechanisms of aging, such as cellular senescence, chronic inflammation, and metabolic disorders, occur in adipose tissue. Therefore, adipose tissue should be considered a significant therapeutic target in antiaging treatments. Emerging interventions against aging targeting adipose tissue have recently been developed. Many studies have demonstrated that reducing the WAT mass and ameliorating WAT dysfunction through many methods, such as exercise, caloric restriction, senolytics and other signaling pathways, can extend health and the lifespan in various organisms (187–195).

### 7.1 Caloric restriction as the foundation of anti-aging therapies

Caloric restriction (CR) without malnutrition has been the foundation of aging for decades (196). A reduction in food intake prolongs the lifespan and delays the onset of age-related diseases in diverse species. The life-prolonging effect of CR is due to changes in many physiological processes, and the biology of AT is closely related (197). As we previously mentioned, hypoxia, mechanical stress and obesity caused by adipocyte hypertrophy are factors contributing to adipose tissue inflammaging. Research has found that the surgical removal of VAT in rats offered approximately 20% of the effect of CR on longevity, preventing insulin resistance and glucose intolerance of aging (189). A reduction in the fat mass, specifically visceral fat, may be a possible underlying mechanism of the antiaging effect of CR (190). However, a recent study has shown that 30% CR alone without fasting or circadian alignment accounts for a 10% extension of the lifespan; however, a daily fasting interval and circadian alignment of feeding act together to extend the lifespan by 35% in male C57BL/6J mice, with improvements in inflammation and immune and metabolic function (198), which are consistent with the results of other recent studies in C57BL/6J male mice (199). Moreover, researchers have found that CR and fasting have overlapping effects on gene expression by performing transcriptomic profiling of inguinal white adipose tissue (iWAT), where CR and fasting altered many Kyoto Encyclopedia of Genes and Genomes (KEGG) pathways, including PPAR, insulin, TGF-β and AMPK signaling and various metabolic pathways (198–201). Many molecules have been discovered to function in regulating the lifespan by dietary restriction; among these, SIRT1, an NAD-dependent deacetylase that participates in cell cycle regulation, is the best established longevity determinant. Studies have shown that SIRT1 mediates the effect of CR on longevity by suppressing lipid accumulation and enhancing adipocyte lipolysis (202, 203). SIRT1 also participates in the regulation of other signaling pathways related to aging. The transactivation of PPARγ, which is essential for proper adipose tissue development and function, is repressed in WAT by SIRT1 (204). Previous studies suggested that experimental Pparg2-deficient mouse models with a lower expression of PPARγ in WAT exhibited a reduction in the lifespan (205). In addition, a new study identified reduced expression of platelet-activating factor acetylhydrolase (PLA2G7) in adipose tissue from people undergoing CR for 2 years by using gene expression profiles, and PLA2G7-deficient mice showed decreased age-related inflammation, lower NLRP3 inflammasome activation, and improved adipose tissue metabolism. These findings demonstrate that PLA2G7 may become an immunometabolic regulator of CR and could potentially be used to lower inflammation and extend the lifespan (206). Moreover, the pathologic expansion of adipose tissue leads to the excessive production of FFA, thereby stimulating TLR4 signaling through the TLR4-NFkB pathway, resulting in the release of proinflammatory cytokines. A study suggested that the expression of three major proinflammatory cytokines (IL-6, MCP1 and TNF-α) in adipose tissue is significantly reduced in old TLR4-KO mice compared to old wild-type mice, showing that TLR4-deficient mice are protected from adipose tissue inflammation during aging (207). Thus, the manipulation of the TLR4 pathway might have great therapeutic potential in aging. The possible molecular pathways described above link caloric restriction to life extension in mammals, providing new insight into the targets of anti-aging treatments.

### 7.2 Senothrerapeutics: senolytics and senomorphics

Over the past decade, the search for strategies that can achieve the beneficial effects of CR without reducing calorie intake has undergone considerable expansion (208). The accumulation of senescent cells (SnCs) is one of the hallmarks of aging, which leads to tissue and organismal aging, and the selective elimination of SnCs in animal models extends the health span (209). Therefore, pharmacological interventions targeting SnCs, also known as senotherapeutics, might be a potential strategy for longevity and the prevention of age-related diseases. Senolytics, drugs that specifically kill SnCs, have shown efficacy against atherosclerosis (210), osteoarthritis (211) and other age-related diseases (212–214). The first senolytics reported by Zhu et al. in 2015 were a drug combination of dasatinib (D), a protein tyrosine kinase inhibitor, and quercetin (Q), a plant flavonoid. By using a transcriptome analysis of senescent and nonsenescent human preadipocytes, the authors revealed that SnCs protect themselves from apoptosis through senescent cell antiapoptotic pathways (SCAPs), including ephrin receptors, BCL-2/BCL-XL family members, P13K/AKT, HIF-1α, etc. (213). *In vivo*, the D + Q combination reduced the senescent cell burden in fat tissue by targeting SCAPs with the benefit of reduced frailty and an extended healthspan (194, 213). To date, D + Q treatment has been tested in several human clinical trials; for example, a clinical trial of D + Q in individuals with diabetic kidney disease found that the D + Q treatment alleviated adipose tissue and the skin senescent cell burden, decreased the resulting adipose tissue macrophage accumulation, enhanced the adipocyte progenitor replicative potential, and reduced key circulating SASP factors (215).

In addition, SnCs can lead to extensive microenvironment dysfunction and cause damage to surrounding cells and tissues due to their proinflammatory SASP (216). Senomorphics, another class of senotherapeutics, is known for modulating the phenotypes of SnCs by interfering with inflammaging, senescence-related signaling pathways, and SASP without inducing apoptosis (217). Resveratrol, a plant-derived polyphenol, is the most potent of the natural SIRT1 activators, and several studies have reported that it can extend the lifespan of various organisms (218–221). In addition to the features of SIRT1 mentioned above, SIRT1 exerts anti-inflammatory activity by inhibiting NF-κB, a key regulator of the immune response and inflammaging (222, 223). Research involving rhesus monkeys fed a high-fat, high-sugar diet suggested that resveratrol improves adipose insulin signaling and reduces the inflammatory response in WAT with increased SIRT 1 expression and decreased NF-κB activation (224). Moreover, SASP in SnCs is regulated by the JAK/STAT pathway, and using the JAK inhibitor roxolitinib in aged mice for 10 weeks reduced both adipose tissue and systemic inflammation and enhanced physical function (225). Metformin, originally approved for the treatment of type 2 diabetes, has been found to have therapeutic effects on age-related diseases, such as insulin resistance, obesity and cardiovascular diseases (226). Numerous studies have proven that metformin is effective in inhibiting cellular senescence and SASPs and preventing age-associated dysfunctions in many model organisms. Metformin modulates aging-related protein synthesis by regulating AMPK/mTOR signaling and enhancing autophagy to increase aging-related protein degradation (227). A recent study demonstrated that metformin reduced cell cycle progression and mTOR signaling and decreased the secretion of most proinflammatory SASP cytokines in mature human adipocytes, exerting anti-inflammatory effects on adipose tissue function (228). Rapamycin, a specific mTOR inhibitor, has been regarded as one of the most well-established senomorphics that reduce cell senescence, suppress SASPs and extend the lifespan. A study focusing on the effects of rapamycin on inflammation in gonadal white adipose tissue (gWAT) of HET3 mice revealed that rapamycin led to a 56% increase in CD45+ leukocytes in gWAT, where the majority of these are ATMs. Interestingly, rapamycin led to an increase in M1 type ATMs, suggesting that rapamycin may achieve life-span extension partially through adipose tissue inflammation (229). L-carnitine, an inhibitor of the JNK/p53 pathway that can prevent apoptosis, has been found to attenuate aging adipose tissue dysfunction by reducing the expression of SASP factors in the WAT of aged (> 18 months old) rats (230).

### 7.3 Immune therapy as an antiaging strategy

Since immune cells play a key role as sources and integrators of inflammatory signals, the regulation of immune cell phenotypes could be a target for intervention to limit ‘inflammaging’ and restore repair capacity in older organisms. Heterochronic parabiosis, a model system in which two animals of different ages are joined to share a common circulatory system, represents an important milestone in aging biology (231). Various circulatory factors have been identified as mediators of the prorejuvenation and proaging systemic effects of heterochronic parabiosis. A recent study demonstrated that transferring eosinophils from young mice reduces WAT and systemic low-grade inflammation, with lower levels of inflammatory factors (such as IL-6, CCL2 and IL-1β), resulting in the restoration of adipose immune homeostasis and widespread rejuvenating consequences for the aging host (125). Another study suggested that adoptive NK-cell infusion reduces senescent markers (p16 and p21) and decreases the SASP phenotype in human adipose tissue (232). Therefore, immune therapy could be a promising strategy for intervention in aging in the future.

### 7.4 Antiaging therapy targeting potential signaling pathways

The JAK/STAT pathway is of great importance in regulating cytokine production and has been investigated as a therapeutic target for many diseases (233–236). Studies have found that the JAK pathway is more highly activated in fat tissue from old than young animals and senescent than nonsenescent cells, and 2 months of administration of ruxolitinib, a specific JAK1/2 inhibitor, reduced systemic inflammation, enhanced physical capacity, preserved fat tissue homeostasis, and improved metabolic function in 22– to 24-month-old mice (237, 238).

As we previously discussed, the p38MAPK pathway also plays a vital role in adipose tissue inflammaging. Studies have proven that the l-arginine-metabolizing enzyme arginase-II (Arg-II) promotes IL-6 production in aging adipose tissues through the p38MAPK pathway. There is more macrophage accumulation in visceral adipose tissues in old WT mice than Arg-II knockout mice. The treatment of aging adipose tissues in WT mice with the specific p38mapk inhibitor SB203580 reduces IL-6 secretion, suggesting that targeting Arg-II or inhibiting p38mapk could be beneficial in reducing age-associated adipose tissue inflammation (239).

In addition, studies have shown that Rolipram is a selective phosphodiesterase 4 (PDE4) inhibitor that activates the AMPK-SIRT6 pathway to reduce adipose deposition and inflammation in aged mice, suggesting that targeting the AMPK-SIRT6 pathway and selective PDE4 inhibitors may be useful agents for the treatment of age-related metabolic dysfunction and diseases (240).

In summary, adipose tissue aging is of great value for studying the basic mechanisms of aging and is an effective therapeutic target for developing new strategies to combat aging and age-related disease (**Table 3**).

**Table 3:** Current antiaging strategies and their potential mechanisms.

Strategies			Potential mechanisms
Caloric restriction			Regulating SIRT1, PPARγ, PLA2G7, TLR4-NFkB pathway etc., to reduce inflammation and improve immune and metabolic function
Senothrerapeutics	Senolytics	Dasatinib (D)+Quercetin (Q)	Targeting SCAPs
	Senomorphics	Resveratrol	SIRT1 activator
		Roxolitinib	JAK inhibitor
		Metformin	Regulating AMPK/mTOR signaling mTOR inhibitor
		Rapamycin	
		L-carnitine	JNK/p53 pathway inhihbitor
Immune therapies	Eosinophils transfer		Reduce inflammation
	Adoptive NK-cell infusion		Reduce senescent markers (p16 and p21) and decreases the SASP phenotype
Antiaging therapy targeting potential signaling pathways	Ruxolitinib Specific p38mapk inhibitor Rolipram		Inhibit JAK/STAT pathway Inhibit p38MAPK pathway Activate AMPK-SIRT6 pathway

## 8 Conclusions and future prospective

Adipose tissue is essential for age-related dysfunction such as metabolic diseases, while aging can also generate multiple effects on adipose tissue, including redistribution of deposits and composition, adipose tissue plasticity reduction, senescent cell accumulation and inflammaging. Among them, adipose tissue inflammation is the most important. This chronic inflammation is usually promoted by senescent/dead cell accumulation, adipocyte hypertrophy, FFA and LPS, and immune cell dysregulation. Various cellular and molecular mechanisms regulate adipose tissue inflammaging. Immune cells are recruited to adipose tissue by different chemokines, and undergo tremendous changes in both their numbers and characteristics during aging. Proinflammatory signaling pathways, including the JAK/STAT, Wnt/β-catenin, NF-κB, and MAPK signaling pathways, control the process of adipose tissue inflammaging in different way. Indeed, Increased inflammaging in aging impacts adipose tissue, leading to adipose tissue dysfunction and ectopic lipid accumulation, further impacting the overall health status. Systemic diseases, such as type II diabetes, CVD and cancer, are somewhat caused by adipose tissue inflammation. Since adipose tissue inflammaging plays pivotal roles, emerging anti-aging interventions have recently been developed targeting adipose tissue. In this review, we summarize the latest approaches that can extend healthy lifespan and delay the onset of age-related diseases including caloric restriction, senothrerapeutics, immune therapies and other strategies targeting adipose tissue inflammaging related signaling pathways. Further research may need to focus on whether suppressing the inflammatory response in adipose tissue can reverse the senescent phenotype, an approach that may identify new targets to relieve aging-associated complications.

## Author contributions

Y-XZ and Z-HY wrote the manuscript and drew the figures. M-YO supervised the manuscript and modified the figures. YS provided a critical review and helped edit the manuscript. S-BZ and Q-FL conceived the idea and supervised the manuscript. All authors contributed to the article and approved the submitted version.
